# Ultra-high-performance supercritical fluid chromatography with quadrupole-time-of-flight mass spectrometry (UHPSFC/QTOF-MS) for analysis of lignin-derived monomeric compounds in processed lignin samples

**DOI:** 10.1007/s00216-017-0663-5

**Published:** 2017-10-13

**Authors:** Jens Prothmann, Mingzhe Sun, Peter Spégel, Margareta Sandahl, Charlotta Turner

**Affiliations:** Department of Chemistry, Centre for Analysis and Synthesis, Lund University, P.O. Box 124, SE-22100 Lund, Sweden

**Keywords:** Column selectivity, Design of experiment, Ionisation efficiency, Lignin, Supercritical fluid chromatography

## Abstract

The conversion of lignin to potentially high-value low molecular weight compounds often results in complex mixtures of monomeric and oligomeric compounds. In this study, a method for the quantitative and qualitative analysis of 40 lignin-derived compounds using ultra-high-performance supercritical fluid chromatography coupled to quadrupole-time-of-flight mass spectrometry (UHPSFC/QTOF-MS) has been developed. Seven different columns were explored for maximum selectivity. Makeup solvent composition and ion source settings were optimised using a D-optimal design of experiment (DoE). Differently processed lignin samples were analysed and used for the method validation. The new UHPSFC/QTOF-MS method showed good separation of the 40 compounds within only 6-min retention time, and out of these, 36 showed high ionisation efficiency in negative electrospray ionisation mode.

**Electronic supplementary material:**

The online version of this article (10.1007/s00216-017-0663-5) contains supplementary material, which is available to authorized users.

**Graphical abstract:**
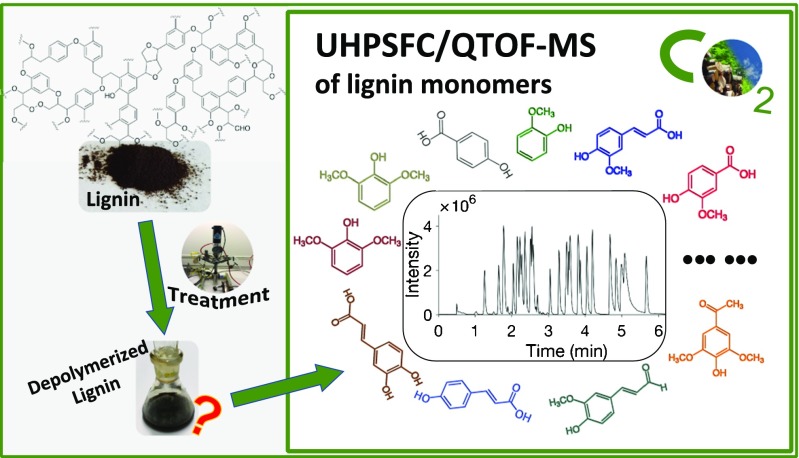
A rapid and selective method for the quantitative and qualitative analysis of 40 lignin-derived compounds using ultra-high-performance supercritical fluid chromatography coupled to quadrupole-time-of-flight mass spectrometry (UHPSFC/QTOF-MS)

## Introduction

Lignin is a complex heterogeneous biopolymer containing differently substituted phenylpropanoid units, with the three basic building blocks being *p*-hydroxyphenyl (H), guaiacyl (G) and syringyl (S) [1]. Lignin is also, by far, the most abundant renewable source of aromatic compounds [2]. A necessary process in successful lignin valorisation is the depolymerisation of lignin into low molecular weight units, which can then be further processed into several value-added compounds [2]. Efficient identification and quantification of lignin-derived compounds in complex mixtures is important for the design of an effective lignin valorisation strategy.

Gas chromatography (GC) with flame ionisation detection (FID) and mass spectrometry (MS) detection has, for long, been the dominating technique for monitoring of lignin depolymerisation [3, 4]. The reasons for this have mainly been the higher resolution, easier coupling with MS and lower costs, compared to LC-based techniques [5]. A disadvantage of GC over LC is the need for a derivatisation step prior to analysis, which will increase the analysis time and also may result in insufficient and discriminative derivatisation that may compromise the quality of the results [6].

In recent years, high-performance LC (HPLC) combined with high-resolution MS (HRMS) has been increasingly applied for the analysis of lignin-derived monomers [7–9]. However, unsatisfying MS sensitivity and in-source fragmentation have been observed with atmospheric pressure ionisation techniques, such as electrospray ionisation (ESI) and atmospheric pressure chemical ionisation (APCI) [9]. The relative ionisation efficiency has been improved for specific lignin products by infusing basic dopants into the mobile phase after the LC column [7–9].

Supercritical fluid chromatography (SFC) uses carbon dioxide (CO_2_) in compressed form as the mobile phase. Compared with liquid mobile phases used in HPLC, supercritical CO_2_ (scCO_2_) has a lower viscosity, allowing for higher flow rates and yielding higher diffusion coefficient for the analytes, which greatly improves the mass transfer [10]. Hence, fast and efficient separation can be achieved by SFC. The resolution in traditional SFC has been further improved by the development of ultra-high-performance supercritical fluid chromatography (UHPSFC) using columns with sub-2-μm packing and the development of modern instrument offering lower dispersion [11]. Both polar and non-polar stationary phases can be used with similar mobile phase compositions [11] and the selectivity tuned by altering the column type, column temperature, backpressure and addition of additives [12, 13]. Compared to GC, SFC has the advantages that no sample derivatisation is needed and the separation can be influenced by altering the mobile phase composition. Currently, SFC has been widely used in the pharmaceutical industry and for analysis of bioactive compounds such as phenolic compounds [14–17]. However, the potential of SFC for analysis of targeted lignin-derived compounds has not yet been comprehensively explored, except for our previous work in which a DAD was used for analysing a relatively small number of lignin-derived compounds [18]. Compared with the HPLC analysis of monomeric lignin model compounds [7, 8, 19], SFC significantly reduced the analysis time.

Compared to mobile phases used in HPLC, the use of highly volatile supercritical CO_2_ in UHPSFC may improve the ionisation efficiency in atmospheric pressure ionisation techniques due to an improved desolvation process [20, 21]. However, analytes may also precipitate before reaching the ion source when the supercritical CO_2_ decompresses. The utilisation of an appropriate makeup solvent may reduce this problem, thereby enhancing transfer of analytes into the ion source. Also, the combination of UHPSFC mobile phase (CO_2_ and co-solvent), makeup solvent and makeup solvent additive influences the desolvation and ionisation of analyte ions [20]. To our knowledge, no optimisation of parameters of importance for the ionisation efficiency has been reported for lignin-derived monomeric compounds using UHPSFC/MS. Grand-Guillaume et al. demonstrated an optimisation of the ionisation efficiency for six pharmaceutical compounds using a UHPSFC/ESI-triple-quadrupole-MS system [22]. Using a design of experiment (DoE) approach, they showed that the mobile phase flow rate and the backpressure have no impact on the ionisation efficiency. However, the makeup solvent flow rate, capillary voltage, desolvation gas temperature and desolvation gas flow rate showed significant effects [22].

In this study, we present, to the best of our knowledge, the first UHPSFC method with HRMS^2^ detection for analysis of lignin-derived monomeric compounds. The selectivities of seven different columns have been studied, along with the influence of makeup solvent compositions and ESI parameters on the ionisation efficiency of a mixture of 40 lignin-derived monomeric compounds. The method has been validated using samples from three different lignin-processing procedures and then finally applied for qualitative and quantitative analysis of four different samples.

## Materials and methods

### Chemicals

Benzoic acid and cinnamic acid were purchased from Mallinckrodt Chemical (Derbyshire UK). The other lignin phenols, as well as ethylvanillin, formic acid, trifluoroacetic acid and ammonium formate, were obtained from Sigma Chemical Co. (St. Louis, MO, USA). Methanol was obtained from Scharlau (Barcelona, Spain). Ethyl acetate and ammonia (2 M solution in methanol) were purchased from Fisher Scientific (Waltham, MA, USA). All organic solvents were of LC-MS grade. All water used was from a Milli-Q Water Purification System with a UV unit.

### Processed lignin samples

Three depolymerised kraft lignin (Indulin AT) samples (samples A, B and C) processed under different conditions were kindly provided by Omar Y. Abdelaziz (Lund University, Lund, Sweden). The kraft lignin was depolymerised under base-catalysed conditions using a continuous plug flow reactor. The samples were dissolved in an aqueous solution with 5 wt% kraft lignin and 5 wt% sodium hydroxide. One depolymerised lignin sample (sample D) was kindly provided by Maxim Galking and Joseph Samec (Stockholm University, Stockholm, Sweden).

### Sample preparation

Three millilitres of the processed lignin sample was acidified to pH 1 with 6 N HCl. Precipitates were removed by centrifugation. The supernatant was collected and extracted three times with 3 mL of ethyl acetate. The ethyl acetate extracts were combined, and the solvent was evaporated under a flow of N_2_. Finally, the solid residue was re-dissolved in 2 mL of methanol.

### Preparation of standards

Single-compound standards with a concentration of 1000 μg/mL were prepared in methanol for guaiacol, eugenol, veratradlehyde, iso-eugenol, syringol, 2,4-dimethylphenol, vanillin, acetovanillone, *o*-cresol, *p*-cresol, phenol, syringaldehyde, acetosyringone, coniferyl aldehyde, benzoic acid, cinnamic acid, 4-methoxybenzoic acid, sinapaldehyde, 3-methoxycinnamic acid, 4-methoxycinnamic acid, 3,5-dimethoxycinnamic acid, *p*-hydroxybenzaldehyde, *p*-hydroxyacetophenone, 3,4-dimethoxycinnamic acid, vanillyl alcohol, coniferyl alcohol, vanillic acid, sinapyl alcohol, syringic acid, 2-(4-hydroxyphenyl)ethanol, ferulic acid, sinapinic acid, guaiacylglycerol-beta-guaiacyl ether, *p*-hydroxybenzoic acid, *p*-coumaric acid, 3,4-dihydroxyhydrocinnamic acid, 3,4-dihydroxyphenylacetic acid, 3,4-dihydroxybenzoic acid, caffeic acid and 3,5-dihydroxybenzoic acid. Standards were further diluted in methanol to a concentration of 250 μg/mL prior to analysis by UHPSFC/quadrupole-time-of-flight (QTOF)-MS. A multi-standard, including all 40 compounds, was prepared by combining 1 mL of each standard followed by evaporation of the solvent under a flow of N_2_. Finally, the dry residue was re-dissolved in 4 mL of methanol to give a final concentration of each compound of 250 μg/mL.

### Equipment

Chromatographic separation was performed with a Waters Ultra Performance Convergence Chromatography System (Waters, Milford, MA, USA) with a diode array detector (ACQUITY UPC^2^ PDA detector, Waters). The UHPSFC/DAD system was also hyphenated via a flow splitter (ACQUITY UPC^2^ splitter, Waters) with a Waters XEVO-G2 QTOF-MS (Waters).

### Software

Instruments were controlled and data acquired using Waters MassLynx 4.1 software. Modde™ 10.1.0 (Umetrics, Umeå, Sweden) was used for creation and evaluation of experimental designs. The open-source software MZmine 2 was used for data evaluation.

### Column and mobile phase additive screening

Seven columns were screened for the separation of the standards on the UHPSFC/DAD: Waters Torus 1-AA (1-aminoanthrocene, 1.7 μm, 3 mm × 100 mm), Torus DIOL (1.7 μm, 3 mm × 100 mm), Torus DEA (diethylamine, 1.7 μm, 3 mm × 100 mm), Torus 2-PIC (2-picolylamine, 1.7 μm, 3 mm × 100 mm), ACQUITY UPC^2^ HSS C18 SB (1.8 μm, 3 mm × 100 mm), ACQUITY UPC^2^ CSH FP (fluorophenyl, 1.7 μm, 3 mm × 100 mm) and ACQUITY UPC^2^ BEH (ethylene-bridged silica, 1.7 μm, 3 mm × 100 mm). The mobile phase consisted of scCO_2_ with methanol as a co-solvent. To improve the peak shape of the relatively more polar phenolic acid analytes, formic acid and ammonium formate were explored as mobile phase additives. In order to compare the selectivity of the columns, similar retention times of the compound test mixture were achieved by using different binary gradient elution programs with solvent A being CO_2_ and solvent B being methanol or methanol with different concentrations of additives. The mobile phase gradient for 1-AA and DIOL columns started at 1.0% B (vol.%), where it was held for 0.5 min and then ramped up to 20% B (vol.%) for 5 min, then held for 2 min and then returning to starting condition in 1 min. The gradient for the 2-PIC column started with 1.0% B (vol.%), held for 0.5 min and then ramped up to 35% B (vol.%) until 6 min, then held for 2 min and decreasing to starting composition in 0.5 min. The mobile phase gradient for the DEA column started with 1.0% B (vol.%), held for 0.5 min and then ramped up to 35% B (vol.%) until 4.5 min, then held for 13.5 min and decreasing to starting composition in 1 min. The mobile phase gradient for the BEH column started with 1.0% B (vol.%), held for 0.5 min and then ramped up to 10% B (vol.%) until 5 min, then held for 2 min and decreasing to starting composition in 1 min. The mobile phase gradient for the C18 and FP columns started with 1.0% B (vol.%), held for 3 min and then ramped up to 10% B (vol.%) until 6 min, then held for 1 min and decreasing to starting composition in 1 min. The flow rate was 2.0 mL/min, the column temperature was 45 °C and the backpressure was 125 bar for all columns. The injection volume was 1.5 μL. The columns were flushed and stored in CO_2_ when not in use. The DAD was collecting data at 20 Hz, the filter time was 0.1 s and spectra from 250 to 500 nm were collected with a resolution of 1.2 nm. Signal data was collected at 280 nm.

### Chromatographic parameters tuning

The DIOL column was chosen for tuning of the chromatographic parameters, as it provided the best overall resolution in a relatively short analysis time. The mobile phase flow rate was varied between 1.5 and 2.5 mL/min, the column temperature was altered between 40 and 60 °C and the backpressure was varied between 110 and 155 bar. Formic acid and ammonium formate were tested as mobile phase additives at different concentrations. One parameter was changed at a time, keeping all other parameters constant (flow rate 2.0 mL/min; column temperature 45 °C; backpressure 125 bar; no mobile phase additive).

*The final optimised UHPSFC method* used the DIOL column at 50 °C as the column temperature and 130 bar as the final backpressure. The elution gradient started with 0% B (vol.%) and then ramped up to 8.5% B (vol.%) until 2.5 min, then ramped up to 25% B (vol.%) until 5.5 min, then held for 2 min and decreasing to starting composition in 0.5 min, with A being CO_2_ and B being methanol. The flow rate was set to 2.0 mL/min, and the injection volume was 1.5 μL.

### Optimisation of mass spectrometer settings

An interaction model with a D-optimal design was used to optimise the MS parameters, using the number of detected peaks in the multi-standard with a relative base peak intensity in negative ionisation mode equal or higher than 1.0*E*5 as a response. The lower limit was set to ensure that a good MS^2^ spectrum could be obtained. Peak detection (using MZmine) was based on exact masses and retention times of the standards. A minimum MS intensity of 1.0*E*5, an *m*/*z* range of ± 0.005 Da and a retention time range of ± 0.05 min were used. A match for exact mass, retention time and MS^2^ spectrum was required for positive identification. Multiple linear regression (MLR) was used to evaluate the D-optimal design. To reduce noise in the model, it was optimised by stepwise removing of insignificant variables and variable interactions until the best cross-validated predictability (*Q* ^2^ *Y*) was reached.

In the D-optimal design, two qualitative and seven quantitative variables were investigated. The two qualitative variables were the type of makeup solvent, methanol or isopropanol, and the type of makeup solvent additive, formic acid, ammonium formate or ammonia (Table 1). To dissolve the ammonium formate, the isopropanol was mixed with 20% methanol. The quantitative variables, makeup solvent flow rate and additive concentration, ESI source temperature, desolvation gas temperature and flow, capillary and cone voltages, were varied as outlined in Table 1. The design included 66 runs with three centre points. The tested values of each experiment are shown in Table S1 (see the Electronic Supplementary Material (ESM)). Analyses were performed in negative ionisation mode, with a cone gas flow of 40 L/h and an extractor cone voltage of 4 V. The scan time was set to 0.1 s with a scan range of *m*/*z* 50–1000. For the optimisation of the MS ionisation efficiency, a previously developed UHPSFC method with the following conditions was used: a BEH 2-EP column (1.7 μm, 3 mm × 100 mm) was used with a column temperature of 45 °C and a backpressure of 125 bar. The elution gradient started with 1% B (vol.%), where it was held for 1 min, followed by a ramp up to 25 % B (vol. %) until 9 min, where it was held for 1 min, after which it returned to starting composition in 1 min, with A being CO_2_ and B methanol. The flow rate was set at 1.0 mL/min. As injection solvent, methanol was used. The injection volume was set to 1.5 μL.

**Table 1:** Overview of the qualitative and quantitative variables for the created design of experiment (D-optimal design) for the optimisation of the MS ionisation efficiency of a mixture of 40 lignin-derived monomeric compounds

	Variable settings	Variable ranges		
		− 1	0	+ 1
Qualitative variables				
Makeup solvents	Methanol, isopropanol			
Makeup solvent additives	Formic acid, ammonium formate, ammonia			
Quantitative variables				
Makeup solvent flow rate (mL/min)		0.2	0.5	0.8
Concentration of makeup solvent additive (mmol/L)		5	10	15
ESI source temperature (°C)		120	135	150
ESI source desolvation gas temperature (°C)		300	450	600
ESI source desolvation gas flow (L/h)		800	1000	1200
ESI source capillary voltage (kV)		2.0	2.5	3.0
ESI source cone voltage (V)		20	35	50

*The best QTOF-MS settings* were as follows: methanol as a makeup solvent, 5 mmol/L ammonia as a makeup solvent additive, a makeup solvent flow rate of 0.2 mL/min, a source temperature of 120 °C, a desolvation gas temperature of 600 °C, a desolvation gas flow of 1200 L/h, a capillary voltage of 3.0 kV and a source cone voltage of 20 V.

### Tandem mass spectrometry experiments

MS^2^ data were collected for each of the standard compounds using the final optimised UHPSFC method combined with the best QTOF-MS settings. A collision-induced dissociation (CID) energy ramp from 20 to 35 V was used.

### Method validation

Spiked processed lignin samples were employed for method validation. One phenolic aldehyde (syringaldehyde), one acid (3,4-dimethoxycinnamic acid) and one alcohol (sinapyl alcohol) were spiked into samples A, B, C and D at 12 different concentrations (0.1, 0.2, 1.0, 2.0, 10, 20, 100, 200, 1000, 2000, 3000 and 5000 μg/mL) to determine the linear dynamic range, limits of detection (LOD) and limits of quantification (LOQ). LOD and LOQ were determined at 3 and 10 times signal-to-noise (S/N) ratio, respectively. Calibration curves for quantitative determination of the three compounds were also drawn based on the results within the dynamic range. The repeatability of the method was examined with six consecutive injections of two spiked samples: one spiked at concentrations near the respective LOQ and the other near the centre of the calibration curve. The reproducibility of the method was examined with injections of the same spiked sample on three non-consecutive days. The recoveries of chromatographic analysis of the three compounds were estimated with the ratio between the slope of the spiked sample calibration curve and that of the calibration curve obtained from injection of standard mixture in the same concentration range.

## Results and discussion

We have previously shown that SFC is a promising technique for the analysis of lignin-derived compounds [18]. Now, we are extending this approach to a broader range of phenolics by exploiting seven different SFC columns with modern stationary phase technology and HRMS detection. A DoE approach was used to optimise the ionisation processes, and the optimised method was validated and applied for the analysis of processed lignin products.

### Column screening

**Fig. 1:**
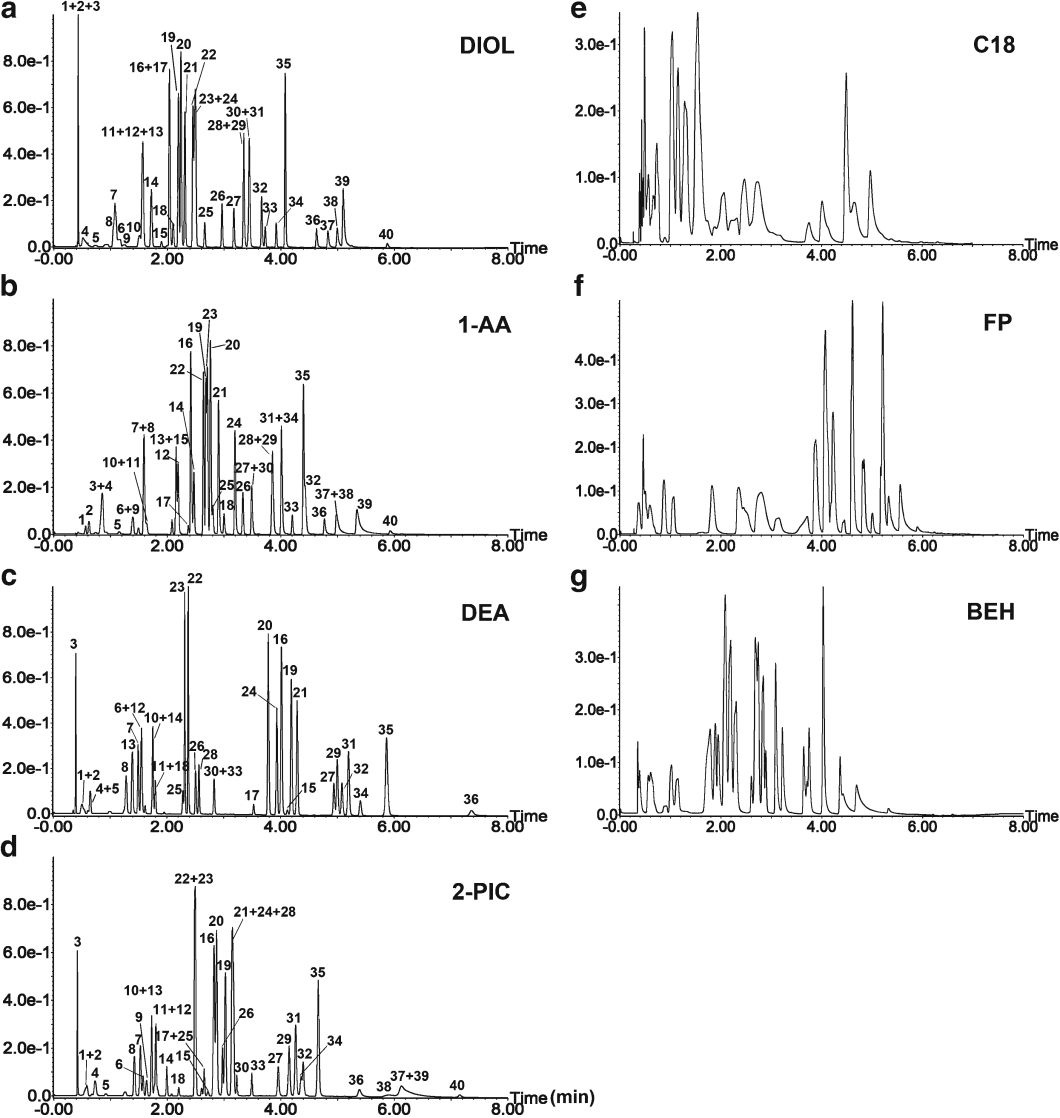
UHPSFC-DAD chromatograms and elution orders of 40 lignin-derived compounds on seven different columns: (**a**) DIOL, (**b**) 1-AA, (**c**) DEA, (**d**) 2-PIC, (**e**) C18, (**f**) FP and (**g**) BEH. For SFC conditions for different columns, see the “Materials and methods” part. For peak identities, see Table 2

**Fig. 2:**
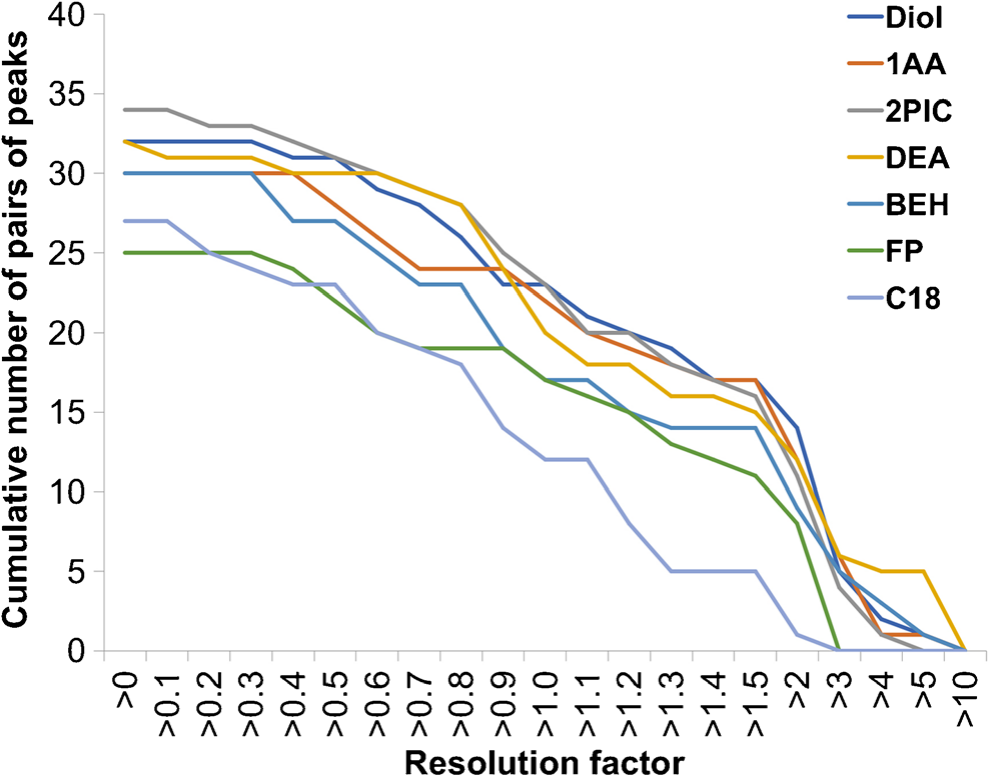
Resolution-level graph for column screening. Different coloured lines represent different columns

As shown in Fig. 1, DIOL, 1-AA, DEA and 2-PIC columns exhibited better resolution than the other three columns: C18, FP and BEH (Fig. 1). The overall resolution achieved for the screened columns is visualised in a resolution-level graph that shows the cumulative number of peaks at resolutions larger than the stated values on the *X*-axis (Fig. 2). The *X*-axis shows a series of different resolution factor levels, while the cumulative number of peaks with a resolution factor qualified for each level is on the *Y*-axis. The graph shows the advantage of DIOL, 1-AA, 2-PIC and DEA columns in terms of both the number of peaks partially separated (0 < Rs < 1.5) and the number of peaks showing baseline separation (Rs > 1.5). The resolving power of these four columns can be attributed to the latest stationary-phase bonding technology and how the chromatographic particles are functionalised. The 1-AA column is the only column among the four that retained and provided reasonable separation of guaiacol, eugenol and veratraldehyde. This is probably due to the enhanced *π*–*π* interactions with the analytes offered by the three benzene rings. Hence, this column can resolve compounds differing in the number of double bonds, for example guaiacol and eugenol (Fig. 1, peaks 1 and 2). Apparent is also the relatively stronger affinity towards guaiacylglycerol-beta-guaiacyl (Fig. 1, peak 33), the only dimeric compound in the mixture, in comparison to the 2-PIC and DEA columns. The dimer was eluted as the 33rd peak, compared to the 22nd peak with DEA and the 28th peak with 2-PIC. However, the 1-AA column provides generally low selectivity for the number of methyl substitutions, as indicated by the co-elution of 2,4-dimethylphenol (Fig. 1, peaks 6 and 9) and *o*-cresol; vanillin and acetovanillone (Fig. 1, peaks 7 and 8); and phenol and *p*-cresol (Fig. 1, peaks 10 and 11).

The high-density DIOL column showed a hydrogen bonding-based separation mechanism. The elution order was largely controlled by the number of hydroxyl and carboxylate groups on the analytes. The dimeric compound guaiacylglycerol-beta-guaiacyl which has three hydroxyl groups showed a strong retention on the DIOL column similarly as with the 1-AA column (eluted as the 33rd peak). Also, the position of the hydroxyl group on the benzene ring contributed to the separation as it decides the availabilities of the hydroxyl groups for interactions.

The 2-PIC column offered some retention of guaiacol and eugenol (Fig. 1, peaks 1 and 2) but failed to separate these two due to relatively weaker *π*–*π* interactions with the analytes compared with the 1-aminoanthrocene column. However, the retention of phenolic acids was stronger. The three most retained acids for both columns were caffeic acid, 3,5-dihydroxybenzoic acid and 3,4-dihydroxyphenylacetic acid. They all had higher retention factors on the 2-PIC column than on the 1-AA column (16.57 compared to 13.23 for 3,4-dihydroxyphenylacetic acid; 16.57 compared to 14.26 for caffeic acid; 19.16 compared to 15.89 for 3,5-dihydroxybenzoic acid). The reason could be that the pyridyl functional group is positively charged which has ion–dipole interaction with the acidic compounds.

The most acidic compounds in the standard mixture, 3,4-dihydroxybenzoic acid, caffeic acid, 3,5-dihydroxybenzoic acid and 3,4-dihydroxyphenylacetic acid (see ESM Fig. S1, peaks 38, 39, 40 and 37), showed very strong retention on the DEA column (*k* = 24.80, 28.03, 28.03 and 32.00, respectively). In fact, they could only be eluted by maintaining a high percentage of methanol in the mobile phase (35 vol.%) for a relatively long time at the end of the gradient. This feature of the column could be attributed to protonated tertiary amines on the stationary phase, resulting in strong ion–dipole interactions with phenolic acids.

The DIOL column was finally selected because of the good general resolution, relatively short analysis time, good peak shapes of acidic compounds and a comparatively lower consumption of co-solvent.

### Tuning of chromatographic parameters

In an attempt to further improve the peak shape of late-eluting peaks on the DIOL column, formic acid and ammonium formate were tested as co-solvent additives at two different concentrations (10 and 50 mM). Formic acid did not improve peak shapes of the late-eluting phenolic acids, while the addition of ammonium formate rather had negative effects (ESM Fig. S2). Although the elution profile was changed with the addition of the additives, no improvement of the overall separation was observed. Interestingly, the addition of formic acid and ammonium formate had opposite impact on retention of the more polar compounds. A possible explanation for this could be that formic acid not only lowers the apparent pH of the mobile phase and eliminates the ionisation of the analytes but also competes with the analytes for binding to the non-endcapped silica sites, resulting in lower retention of the analytes [23, 24]. The addition of ammonium formate might help to stabilise the apparent pH of the mobile phase, which might cause some acidic compounds to be in ionised form. Additionally, ammonium formate adsorbed on the stationary surface and in the layer covering the stationary phase with higher co-solvent concentration than the bulk mobile phase can increase acidic analyte adsorption on the stationary phase through ion pairing [25].

The selectivity of the column was unaffected by the mobile phase flow rate. The best overall separation was achieved at 2 mL/min, the same flow rate used in the column screening experiment, with lower flow rates causing unnecessarily lengthened analysis time without any obvious improvement in resolution and higher flow rates showing negative effect on resolution (Fig. S3).

As expected, raising the backpressure while keeping other parameters constant generally resulted in weaker retention and shorter analysis time, due to increased elution strength caused by an increase in mobile phase density (ESM Fig. S4). This effect was insignificant for the late-eluting compounds, due to the lower compressibility of the mobile phase containing a large fraction of methanol. Temperature increase at the same backpressure (125 bar) also lowered the mobile phase density, which accordingly increased the retention of the analytes (Fig. S5).

The final method had a column temperature of 50 °C, a backpressure of 130 bar and a flow rate of 2.0 mL/min. The elution order of all analytes follows the order of polarity in general with acidic analytes being retained the most compared to ketones and aldehydes. Like what was discussed in the column screening part, for compounds with similar structure and polarity, the number of polar moieties and their availability determine the order of elution.

Compared with previously reported HPLC methods of various sets of monomeric lignin model compounds [7, 8, 19], our method greatly shortened the analysis time with a significantly enriched set of compounds. Another advantage shown by the SFC-MS method is the separation of isomeric compounds. Eleven major lignin-derived phenols from alkaline CuO oxidation were analysed with UHPLC in 15 min [26]. In our study, a larger set of compounds including the aforementioned 11 phenols was analysed with most of them separated in a shorter time (6 min). Three lignin monomers in wheat straw were analysed with UHPLC in 3 min [27] in which the three peaks eluted close to each other between 2.4 and 2.9 min. In comparison, our method had longer analysis time but separating more compounds. Also, the peaks were well scattered among the whole elution range.

### Mass spectrometry method optimisation

**Fig. 3:**
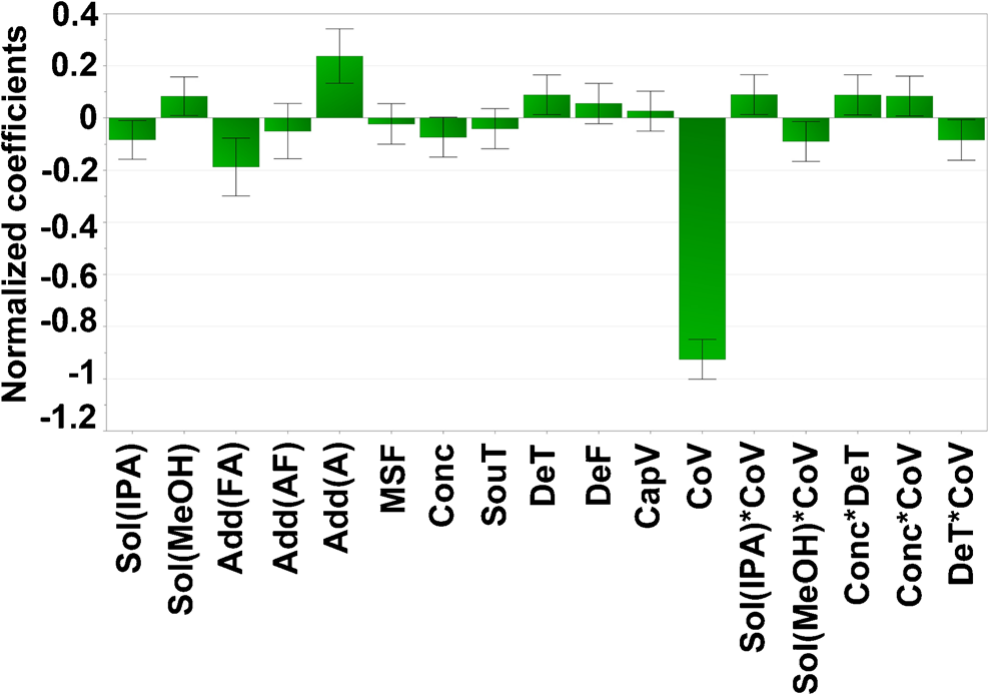
Normalised influence of investigated variables on the number of detected peaks with a base peak intensity ≥ 1.0*E*5. Sol solvent, Add additive, IPA isopropanol, MeOH methanol, FA formic acid, AF ammonium formate, A ammonia, MSF makeup solvent flow rate, Conc concentration of makeup solvent additive, SouT ion source temperature, DeT desolvation gas temperature, DeF desolvation gas flow, CapV capillary voltage, CoV cone voltage

To optimise the MS ionisation efficiency, the influence of two qualitative and seven quantitative variables was investigated using a D-optimal design using the number of peaks detected as response. The optimised model showed a total explained variance of 93% [*R* ^2^(*Y*) = 0.93] and a cross-validated predictability of 89% [*Q* ^2^(*Y*) = 0.89]. The normalised influence of all significant variables and variable interactions is shown in Fig. 3. The correlation between the predicted number of detected peaks versus the observed number of detected peaks is shown in Fig. S6 (see ESM). The predicted versus experimental data were analysed by linear regression. The experimental and predicted values are highly correlated (*p* < 0.001) and did not differ from the ideal line with a slope of one. From the investigated makeup solvents and makeup solvent additives, only methanol and ammonia respectively showed a positive influence on the response. Therefore, the combination of methanol and ammonia was determined to be the optimal solvent–additive combination. Isopropanol and formic acid showed a negative influence on the response, while ammonium formate showed no significant effect. From the investigated quantitative variables, the desolvation gas temperature showed a significant positive influence and the cone voltage a significant negative influence. The positive influence of the desolvation gas temperature could be explained by a more efficient desolvation process at higher temperatures. The negative influence of the cone voltage most likely resulted from increased fragmentation of the standard compounds at higher cone voltages. The concentration of the makeup solvent additive did not show a significant effect, although it did interact significantly with both cone voltage and desolvation gas temperature. The makeup solvent flow rate, source temperature, desolvation gas flow rate and capillary voltage did not influence the response.

Next, an optimiser function, based on a simplex algorithm with a non-linear desirability function, was used to determine factor settings resulting in the highest number of peaks with a relative MS intensity of ≥ 1.0*E*5. The optimal response was found with methanol as a makeup solvent, 5 mmol/L ammonia as a makeup solvent additive, a makeup solvent flow rate of 0.2 mL/min, a source temperature of 120 °C, a desolvation gas temperature of 600 °C, a desolvation gas flow of 1200 L/h, a capillary voltage of 3.0 kV and a cone voltage of 20 V.

**Fig. 4:**
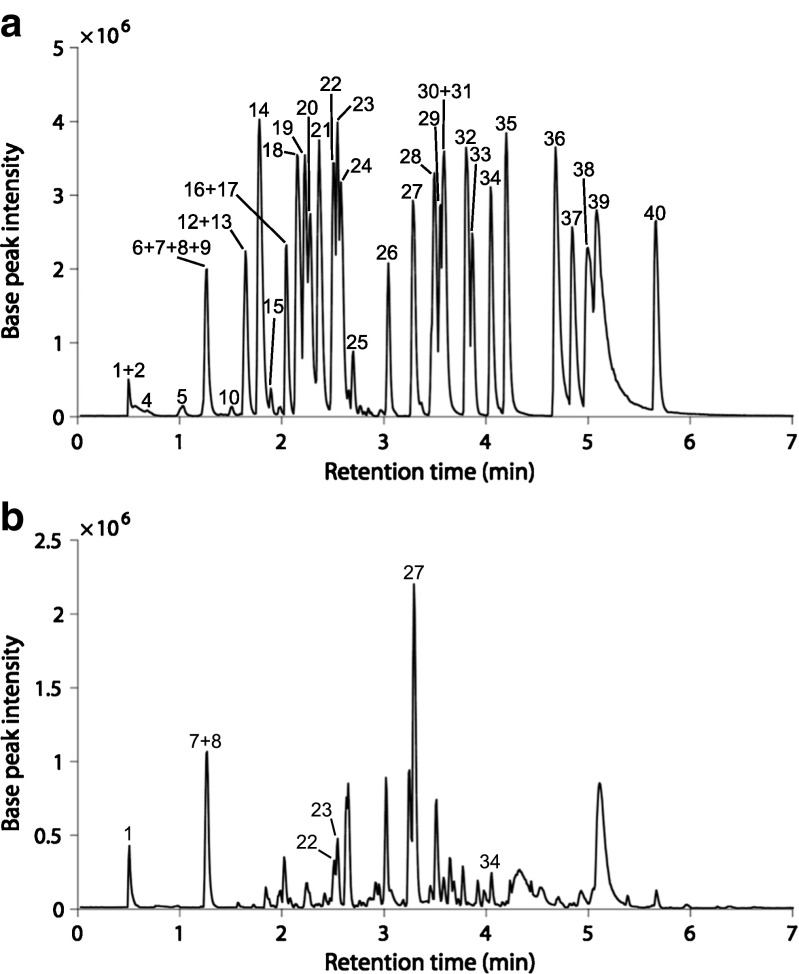
Obtained base peak ion chromatogram of (**a**) the multi-standard including 40 lignin-related compounds and (**b**) lignin sample A, using the optimised UHPSFC/QTOF-MS conditions. For peak identities of the multi-standard, see Table 1. Identified compounds in sample A with the use of the multi-standard are labeled according to the compound number in Table 2

Finally, the optimised condition was tested, enabling the detection of 36 out of 40 compounds in the multi-standard in a single analysis with a relative base peak intensity ≥ 1.0*E*5 (Table 2, Fig. 4). Only iso-eugenol (relative base peak intensity 9.5*E*4), phenol (relative MS intensity 3.1*E*4) and 3,4-dihydroxyphenylacetic acid (relative base peak intensity 9.4*E*4) were below the base peak intensity threshold. Veratraldehyde could not be detected under these conditions.

**Table 2:** Measured retention times, calculated and measured *m*/*z* values, base peak intensities and obtained MS^2^ fragments of the 40 lignin-derived monomeric compounds in the multi-standard. MS^2^ fragments are evaluated from single-standard runs

Peak number	Compound	Retention time (min)	Calculated *m*/*z* ([M-H]^−^)	Measured *m*/*z* ([M-H]^−^)	Base peak intensity	MS^2^ fragmentation	
						MS^2^ transition	Lost fragment
1	Guaiacol	0.48	123.0446	123.0440	1.4*E*5	123 → 108 123 → 75	CH_3_ H_2_O + OCH_2_
2	Eugenol	0.48	163.0759	163.0755	6.0*E*5	163 → 149 163 → 148 163 → 121	CH_2_ CH_2_ + CH_3_ C_2_H_3_ + CH_3_
3	Veratraldehyde	0.48^a^	165.0552	–	–	–	–
4	Iso-eugenol	0.67	163.0759	163.0757	9.6*E*4	163 → 149 163 → 148 163 → 121	CH_2_ CH_2_ + CH_3_ C_2_H_3_ + CH_3_
5	Syringol	1.02	153.0552	153.0546	1.6*E*5	153 → 123 153 → 95	2× CH_3_ 2× CH_3_ + CO
6	2,4-Dimethylphenol	1.23	121.0654	121.0648	2.6*E*5	–	–
7	Vanillin	1.26	151.0395	151.0391	2.1*E*6	151 → 136 151 → 108 151 → 92	CH_3_ CH_3_ + CO CH_3_ + CO + O
8	Acetovanillone	1.26	165.0552	165.0543	2.0*E*6	165 → 150 165 → 122	CH_3_ CH_3_ + CO
9	*o*-Cresol	1.29	107.0497	107.0492	1.5*E*5	–	–
10	*p*-Cresol	1.50	107.0497	107.0493	1.3*E*5	107 → 91 107 → 75	O OH + CH_3_
11	Phenol	1.56	93.0341	93.0340	3.2*E*4	–	–
12	Syringaldehyde	1.64	181.0501	181.0498	2.3*E*6	181 → 166 181 → 151 181 → 123	CH_3_ OCH_2_ 2× CH_3_ + CO
13	Acetosyringone	1.63	195.0658	195.0652	1.6*E*6	195 → 180 195 → 165 195 → 137	CH_3_ 2× CH_3_ 2× CH_3_ + CO
14	Coniferyl aldehyde	1.77	177.0552	177.0545	4.1*E*6	177 → 162 177 → 134	CH_3_ CH_3_ + CO
15	Benzoic acid	1.89	121.0290	121.0287	3.8*E*5	121 → 77^b^	CO_2_
16	Cinnamic acid	2.04	147.0446	147.0446	2.3*E*6	147 → 103 147 → 77	CO_2_ C_2_H_2_CO_2_
17	4-Methoxybenzoic acid	2.06	151.0395	151.0393	8.1*E*6	151 → 107 151 → 92	CO_2_ CO_2_ + CH_3_
18	Sinapaldehyde	2.14	207.0658	207.0654	3.4*E*6	207 → 177 207 → 149	2× CH_3_ 2× CH_3_ + CO
19	3-Methoxycinnamic acid	2.22	177.0552	177.0548	3.6*E*6	177 → 133 177 → 103	CO_2_ OCH_2_ + CO_2_
20	4-Methoxycinnamic acid	2.27	177.0552	177.0547	2.8*E*6	177 → 133 177 → 117	CO_2_ C_2_H_2_CO_2_
21	3,5-Dimethoxycinnamic acid	2.36	207.0658	207.0655	3.5*E*6	207 → 133 207 → 118	CO_2_ + CH_2_O CO_2_ + CH_3_ + CH_2_O
22	*p*-Hydroxybenzaldehyde	2.50	121.0290	121.0287	3.5*E*6	121 → 92	CHO
23	*p*-Hydroxyacetophenone	2.54	135.0446	135.0441	4.0*E*6	135 → 120 135 → 92	CH_3_ C_2_H_3_O
24	3,4-Dimethoxycinnamic acid	2.57	207.0658	207.0650	3.0*E*6	207 → 103	CO_2_ + 2× CH_2_O
25	Vanillyl alcohol	2.69	153.0552	153.0550	8.2*E*5	153 → 135 153 → 120	H_2_O H_2_O + CH_3_
26	Coniferyl alcohol	3.03	179.0708	179.0705	2.0*E*6	179 → 164 179 → 146	CH_3_ CH_3_ + H_2_O
27	Vanillic acid	3.28	167.0345	167.0336	2.9*E*6	167 → 152 167 → 123 167 → 108	CH_3_ CO_2_ CH_3_ + CO_2_
28	Sinapyl alcohol	3.46	209.0814	209.0808	1.2*E*6	209 → 179 209 → 161 209 → 151	2× CH_3_ 2× CH_3_ + H_2_O 2× CH_3_ + CO
29	Syringic acid	3.50	197.0450	197.0446	3.4*E*6	197 → 167 197 → 123 197 → 95	2× CH_3_ 2× CH_3_ + CO_2_ 2× CH_3_ + CO_2_ + CO
30	2-(4-Hydroxyphenyl)ethanol	3.55	137.0603	137.0594	2.9*E*6	137 → 119 137 → 106	H_2_O CH_2_OH
31	Ferulic acid	3.58	193.0501	193.0497	3.7*E*6	193 → 134	CH_3_ + CO_2_
32	Sinapinic acid	3.81	223.0607	223.0603	3.7*E*6	223 → 193 223 → 149 223 → 121	2× CH_3_ 2× CH_3_ + CO_2_ 2× CH_3_ + CO_2_ + 2× CH_2_
33	Guaiacylglycerol-beta-guaiacyl ether	3.87	319.1182	319.1174	5.9*E*5	319 → 256 319 → 149	CH_2_O + H_2_O + CH_3_ CH_2_O + H_2_O + CH_3_ + C_6_H_3_O_2_
34	*p*-Hydroxybenzoic acid	4.05	137.0239	137.0245	3.0*E*6	137 → 93	CO_2_
35	*p*-Coumaric acid	4.19	163.0395	163.0390	3.8*E*6	163 → 119 163 → 93	CO_2_ C_2_H_2_CO_2_
36	3,4-Dihydroxyhydrocinnamic acid	4.66	181.0501	181.0492	3.6*E*6	181 → 137	CO_2_
37	3,4-Dihydroxyphenylacetic acid	4.84	167.0345	167.0345	9.0*E*4	167 → 123	CO_2_
38	3,4-Dihydroxybenzoic acid	4.98	153.0188	153.0177	2.3*E*6	153 → 109	CO_2_
39	Caffeic acid	5.07	179.0345	179.0342	2.7*E*6	179 → 135	CO_2_
40	3,5-Dihydroxybenzoic acid	5.66	153.0188	153.0186	2.9*E*6	153 → 109	CO_2_

^a^Retention time from UV detection

^b^Fragment seen in MS^1^ spectrum

Because of the high influence of the cone voltage on the response, the cone voltage 20 V might not be the optimal value. To investigate the influence of the cone voltage, the concentration of the makeup solvent additive and the desolvation gas temperature in more detail, a second design of experiment has been performed. A quadratic model with a face-centred central composite design (CCF) including 17 runs with three centre points was used. However, the results showed no significant improvement compared to the first design of experiment. The obtained results lead to the conclusion that the optimal value for the cone voltage is close to 20 V. The experimental design and the results of the second design of experiment are shown in the supporting information (see ESM, Tables S2 and S3, Figs. S7 and S8).

### Method validation

LOD, LOQ, dynamic range and within-day and inter-day precision of peak area for the three compounds (syringaldehyde, 3,4-dimethoxycinnamic acid and sinapyl alcohol) in lignin samples A, B and C are shown in Table 3. The higher LOD and LOQ observed in the C sample can be attributed to its greater complexity as compared with the other two samples, causing increased matrix interference and ionisation suppression.

**Table 3:** Obtained results of limit of detection (LOD), limit of quantification (LOQ), linear dynamic range, repeatability and reproducibility of syringaldehyde, 3,4-dimethoxycinnamic acid and sinapyl alcohol from method validation with spiked samples

Lignin sample	Compound spiked	LOD in μg/mL	LOQ in μg/mL	Dynamic range in μg/mL	Repeatability, % (μg/mL)		Reproducibility, % (μg/mL)	
					RSD near LOQ	RSD near middle of dynamic range	RSD near LOQ	RSD near middle of dynamic range
A	Syringaldehyde	1.0	2.0	2.0–1000	5.2 (10)	1.6 (400)	17.0 (10)	11.7 (400)
	3,4-Dimethoxycinnamic acid	0.2	1.0	1.0–200	2.6 (5.0)	1.7 (100)	9.1 (5.0)	11.3 (100)
	Sinapyl alcohol	1.0	2.0	2.0–1000	11.2 (10)	1.7 (400)	9.2 (10)	7.2 (400)
B	Syringaldehyde	1.0	2.0	2.0–1000	3.2 (10)	3.5 (400)	15.0 (10)	15.1 (400)
	3,4-Dimethoxycinnamic acid	0.2	1.0	1.0–200	3.8 (5.0)	1.9 (100)	12.2 (5.0)	10.0 (100)
	Sinapyl alcohol	1.0	2.0	2.0–1000	3.1 (10)	2.2 (400)	10.3 (10)	8.1 (400)
C	Syringaldehyde	2.0	20	20–1000	2.2 (20)	6.6 (400)	18.7 (20)	3.5 (400)
	3,4-Dimethoxycinnamic acid	1.0	1.5	1.5–400	6.6 (1.5)	2.7 (200)	18.7 (1.5)	10.6 (200)
	Sinapyl alcohol	2.0	10	10–2000	1.8 (10)	1.9 (1000)	19.6 (10)	16.2 (1000)

RSD: relative standard deviation

The impact of sample matrix effects on target analyte signal intensity was evaluated by comparing the slopes of calibration curves derived from pure standards and from spiked samples [28]. As can be calculated from the results shown in Table 4, syringaldehyde showed a 46% decrease in sample A. Calibration curve slopes for 3,4-dimethoxycinnamic acid and sinapyl alcohol in sample A were not impacted by the matrix. In B samples, the calibration curve slope for sinapyl alcohol was unaltered, whereas the slopes were 36 and 17% lower for syringaldehyde and 3,4-dimethoxycinnamic acid, respectively, and the calibration curve slope decreased for all spiked compounds in sample C (80% for syringaldehyde, 42% for 3,4-dimethoxycinnamic acid and 12% for sinapyl alcohol). Hence, the greater complexity of the C sample also led to a more pronounced ionisation suppression. As a consequence of these matrix effects, the quantitative analysis based on the calibration obtained with pure standards is rendered unreliable.

**Table 4:** Obtained coefficients of determination (*R* ^2^) and calibration curve slopes in the dynamic range of syringaldehyde, 3,4-dimethoxycinnamic acid and sinapyl alcohol for spiked samples and standard mixtures

Sample	Compound spiked	*R* ^2^		Calibration curve slopes	
		Spiked sample	Standard mixture	Spiked sample	Standard mixture
A	Syringaldehyde	0.9999	0.9920	50.64 ± 0.21	92.94 ± 4.17
	3,4-Dimethoxycinnamic acid	0.9958	0.9792	291.62 ± 9.44	291.24 ± 21.24
	Sinapyl alcohol	0.9922	0.9987	56.30 ± 2.50	53.87 ± 0.97
B	Syringaldehyde	0.9985	0.9920	60.00 ± 1.15	92.94 ± 4.17
	3,4-Dimethoxycinnamic acid	0.9950	0.9792	303.91 ± 10.83	291.24 ± 21.24
	Sinapyl alcohol	0.9986	0.9987	65.38 ± 1.24	53.87 ± 0.97
C	Syringaldehyde	0.9971	0.9926	18.92 ± 0.73	90.84 ± 5.55
	3,4-Dimethoxycinnamic acid	0.9866	0.9726	214.47 ± 11.20	320.87 ± 24.09
	Sinapyl alcohol	0.9999	0.9740	36.09 ± 0.15	40.98 ± 3.35

The MS ionisation efficiency can be compared using the achieved LODs. Owen et al. achieved for similar lignin-related monomers lower LODs compared to this study by using reversed-phase LC coupled to a linear ion trap/Fourier transform ion cyclotron resonance MS (vanillin 4.0 × 10^−3^ μg/mL; 2-methoxy-4-propylphenol 3.0 × 10^−3^ μg/mL) [8]. Also, Zheng et al. achieved lower LODs by using reversed-phase LC coupled to a triple-quadrupole MS (*p*-hydroxybenzaldehyde 4.0 × 10^−4^ μg/mL; vanillin 2.1 × 10^−4^ μg/mL; syringaldehyde 8.9 × 10^−4^ μg/mL) [27]. A possible reason might be that the LODs determined in this study (Table 3) have been determined by using spiked lignin samples instead of solvent standards like in the studies of Owen et al. and Zhang et al. Using spiked lignin samples gives ion suppression effects and negative effects on the LOD. The use of different mass analysers also explains the deviating LODs.

### Analysis of processed lignin samples

A qualitative analysis of the processed lignin samples was performed with the optimised UHPSFC/QTOF-MS method (see Table 5). Several lignin-derived monomeric compounds could be identified. A base peak ion chromatogram of sample A is shown in Fig. 4. The base peak ion chromatograms of samples B, C and D are shown in Fig. S9 (see ESM). In samples A, B and C, vanillin, acetovanillone and *p*-hydroxyacetophenone could be identified. Guaiacol and *p*-hydroxybenzaldehyde could be identified in sample A as well as in sample C, and vanillic acid and *p*-hydroxybenzoic acid could be identified in sample A and sample B. In sample D, sinapaldehyde, syringic acid, ferulic acid and sinapinic acid could be identified. The base peak ion chromatogram of all lignin samples shows that the samples are complex mixtures and include more compounds than the identified ones. Further work is required to identify these unknown peaks.

**Table 5:** Identified compounds in the processed lignin samples

Sample	Compound	Retention time (min)	Calculated *m*/*z* ([M-H]^−^)	Measured *m*/*z* ([M-H]^−^)	Base peak intensity	MS^2^ fragmentation	
						MS^2^ transition	Lost fragment
A	Guaiacol	0.51	123.0446	123.0446	4.3*E*5	123 → 108	CH_3_
	Vanillin	1.27	151.0395	151.0397	5.1*E*5	151 → 136 151 → 108	CH_3_ CH_3_ + CO
	Acetovanillone	1.27	165.0552	165.0556	1.1*E*6	165 → 150 165 → 122	CH_3_ CH_3_ + CO
	*p*-Hydroxybenzaldehyde	2.51	121.0290	121.0293	3.3*E*5	121 → 92	CHO
	*p*-Hydroxyacetophenone	2.55	135.0446	135.0445	4.8*E*5	135 → 120 135 → 108	CH_3_ C_2_H_3_
	Vanillic acid	3.29	167.0345	167.0356	2.2*E*6	167 → 152 167 → 123 167 → 108	CH_3_ CO_2_ CH_3_ + CO_2_
	*p*-Hydroxybenzoic acid	4.05	137.0239	137.0251	2.5*E*5	137 → 93	CO_2_
B	Vanillin	1.27	151.0395	151.0399	2.3*E*5	151 → 136 151 → 108	CH_3_ CH_3_ + CO
	Acetovanillone	1.27	165.0552	165.0563	1.7*E*5	165 → 150 165 → 122	CH_3_ CH_3_ + CO
	*p*-Hydroxyacetophenone	2.55	135.0446	135.0447	1.6*E*5	135 → 120 135 → 108	CH_3_ C_2_H_3_
	Vanillic acid	3.29	167.0345	167.0349	2.3*E*6	167 → 152 167 → 123 167 → 108	CH_3_ CO_2_ CH_3_ + CO_2_
	*p*-Hydroxybenzoic acid	4.05	137.0239	137.0254	2.4*E*5	137 → 93	CO_2_
C	Guaiacol	0.50	123.0446	123.0442	1.4*E*6	123 → 108	CH_3_
	Vanillin	1.26	151.0395	151.0391	3.0*E*5	151 → 136 151 → 108	CH_3_ CH_3_ + CO
	Acetovanillone	1.26	165.0552	165.0553	1.7*E*6	165 → 150 165 → 122	CH_3_ CH_3_ + CO
	*p*-Hydroxybenzaldehyde	2.49	121.0290	121.0290	1.2*E*5	121 → 92	CHO
	*p*-Hydroxyacetophenone	2.53	135.0446	135.0441	8.2*E*5	135 → 120 135 → 108	CH_3_ C_2_H_3_
D	Sinapaldehyde	2.14	207.0658	207.0645	1.6*E*4	207 → 192 207 → 177	CH_3_ 2× CH_3_
	Syringic acid	3.50	197.0450	197.0446	3.4*E*6	197 → 123 197 → 95	2× CH_3_ + CO_2_ 2× CH_3_ + CO_2_ + CO
	Ferulic acid	3.60	193.0501	193.0490	1.5*E*4	193 → 134	CH_3_ + CO_2_
	Sinapinic acid	3.81	223.0607	223.0603	3.7*E*6	223 → 193 223 → 149 223 → 121	2× CH_3_ 2× CH_3_ + CO_2_ 2× CH_3_ + CO_2_ + 2× CH_2_

## Conclusions

An UHPSFC/QTOF-MS method for the analysis of lignin-derived monomeric compounds from processed lignin samples was developed in this work. Thirty-two of the 40 compounds are partially or baseline separated in less than 6 min. Column screening results showed that the bonding technology and functionalisation of chromatographic particles contribute to a significant improvement in resolving power. Enhanced *π*–*π* interaction is needed to separate the early eluting lignin phenols with similar structures. Hydrogen bonding interaction can be effectively harnessed to separate lignin-derived compounds. However, ion–ion interaction is not appreciated as it lengthens the retention time of phenolic acids to an unnecessary extent. With the optimised MS method, 36 of 40 compounds could be detected with a relative MS intensity of ≥ 1.0*E*5. The cone voltage and the desolvation temperature were found to have the strongest impact of all investigated ion source parameters on ionisation efficiency. Finally, the applicability of the method for qualitative and quantitative analysis of processed lignin samples was demonstrated. Clearly, UHPSFC-MS has its benefits in comparison to UHPLC-MS in terms of selectivity and speed.

## Electronic supplementary material

ESM 1 (PDF 3044 kb)
