# Supplementary material for: Ultra-high-performance supercritical fluid chromatography with quadrupole-time-of-flight mass spectrometry (UHPSFC/QTOF-MS) for analysis of lignin-derived monomeric compounds in processed lignin samples
Source: Anal Bioanal Chem. 2017 Oct 13;409(30):7049–61. doi: 10.1007/s00216-017-0663-5 (PMC5717129; doi:10.1007/s00216-017-0663-5)
Supplement: Supplementary file 1 — (PDF 3044 kb) [file 216_2017_663_MOESM1_ESM.pdf]

Analytical and Bioanalytical Chemistry

Electronic Supplementary Material

Ultra-high-performance supercritical fluid chromatography with quadrupole-time-of-flight mass spectrometry (UHPSFC/QTOF-MS) for analysis of lignin-derived monomeric compounds in processed lignin samples

Jens Prothmann, Mingzhe Sun, Peter Spégel, Margareta Sandahl, Charlotta Turner

Content

Table S1	Performed experiments of the D-optimal interaction model design
Figure S1	Full UHPSFC-DAD chromatogram of 40 lignin-derived compounds on DEA column
Figure S2	The influence of different additives (formic acid and ammonium formate) to the mobile phase at different concentrations
Figure S3	The influence of different flow rates
Figure S4	The influence of different backpressure
Figure S5	The influence of different column temperatures
Figure S6	Correlation between predicted and observed number of detected peaks with base peak intensity ≥1.0E5 of the D-optimal interaction model design
Table S2	Overview of the quantitative variables for the second created design of experiment (face centered central composite design) for the more detailed investigation of the influence of the concentration of the make-up solvent additive, the desolvation gas temperature and the cone voltage on the MS ionisation efficiency of a mixture of 40 lignin-derived monomeric compounds
Table S3	Performed experiments of the second design of experiment (quadratic model with face centered central composite design)
Figure S7	Normalized influence of the investigated variables on the number of detected peaks with a base peak intensity ≥1.0E5 of the second design of experiment (quadratic model with face centered central composite design)
Figure S8	Correlation between predicted and observed number of detected peaks with base peak intensity ≥1.0E5 of the second design of experiment (quadratic model with face centered central composite design)
Figure S9	Obtained base peak ion chromatogram for (a) lignin sample B; (b), lignin sample C; and (c) lignin sample D, using the optimized UHPSFC-QTOF/MS conditions

Table S1 Performed experiments of the D-optimal interaction model design. For variable ranges see Table 1. Exp No = experiment number; Sol = solvent; Add = additive; IPA = isopropanol; MeOH = ethanol; FA = formic acid; AF = ammonium formate; A = ammonia; MSF = make-up solvent flow rate; Conc = concentration of make-up solvent additive; SouT = ion source temperature; DeT = desolvation gas temperature; DeF = desolvation gas flow; CapV = capillary voltage; CoV = cone voltage; BPI = base peak ion chromatogram

Exp No	Run Order	Sol	Add	MSF	Conc	SouT	DeT	DeF	CapV	CoV	Peaks with BPI intensity ≥1.0E5
1	43	IPA	FA	-1	-1	1	1	-1	-1	-1	18
2	22	IPA	FA	-1	1	1	-1	1	-1	-1	10
3	1	IPA	FA	1	1	1	-1	-1	1	-1	12
4	16	IPA	FA	1	-1	-1	-1	1	1	-1	18
5	6	IPA	FA	-1	1	-1	-1	-1	-1	1	1
6	29	IPA	FA	1	-1	1	-1	-1	-1	1	2
7	60	IPA	FA	1	1	1	1	1	-1	1	2
8	31	IPA	FA	1	1	-1	1	-1	1	1	2
9	42	IPA	FA	-1	-1	1	-1	1	1	1	2
10	46	MeOH	FA	1	1	-1	1	-1	-1	-1	23
11	20	MeOH	FA	-1	-1	1	-1	1	-1	-1	23
12	10	MeOH	FA	-1	-1	-1	-1	-1	1	-1	25
13	49	MeOH	FA	-1	1	-1	1	1	1	-1	27
14	41	MeOH	FA	1	-1	1	1	1	1	-1	20
15	35	MeOH	FA	-1	1	1	1	-1	-1	1	2
16	28	MeOH	FA	-1	-1	-1	1	1	-1	1	2
17	62	MeOH	FA	1	-1	-1	1	-1	1	1	2
18	25	MeOH	FA	1	1	-1	-1	1	1	1	4
19	3	IPA	AF	1	-1	1	-1	-1	-1	-1	21
20	65	IPA	AF	-1	-1	-1	-1	1	-1	-1	25
21	47	IPA	AF	1	1	-1	1	1	-1	-1	22
22	54	IPA	AF	1	-1	-1	1	-1	1	-1	22
23	18	IPA	AF	1	1	1	-1	1	1	-1	20
24	58	IPA	AF	-1	-1	1	1	1	1	-1	27
25	51	IPA	AF	1	-1	-1	1	-1	-1	1	2
26	36	IPA	AF	1	1	-1	-1	1	-1	1	2
27	52	IPA	AF	-1	1	1	1	1	-1	1	2
28	64	IPA	AF	-1	1	1	-1	-1	1	1	2
29	12	IPA	AF	-1	-1	-1	1	1	1	1	4
30	55	IPA	AF	1	-1	1	1	1	1	1	2
31	30	MeOH	AF	-1	1	1	-1	-1	-1	-1	18
32	61	MeOH	AF	-1	-1	-1	1	-1	-1	-1	24
33	11	MeOH	AF	1	-1	1	1	1	-1	-1	26
34	59	MeOH	AF	1	1	-1	-1	-1	1	-1	17
35	15	MeOH	AF	1	1	1	1	-1	1	-1	20
36	45	MeOH	AF	1	-1	-1	1	1	1	-1	25
37	21	MeOH	AF	1	-1	-1	-1	1	-1	1	2
38	32	MeOH	AF	-1	1	-1	-1	1	-1	1	3
39	48	MeOH	AF	1	1	1	-1	1	-1	1	2
40	26	MeOH	AF	1	-1	1	-1	-1	1	1	2

41	13	MeOH	AF	-1	1	-1	1	-1	1	1	2
42	37	MeOH	AF	-1	-1	1	-1	1	1	1	2
43	40	IPA	A	-1	1	-1	-1	-1	-1	-1	9
44	9	IPA	A	1	1	1	1	-1	-1	-1	26
45	7	IPA	A	1	-1	-1	1	1	-1	-1	28
46	14	IPA	A	-1	-1	1	-1	-1	1	-1	30
47	17	IPA	A	1	1	-1	1	1	1	-1	27
48	38	IPA	A	-1	1	-1	1	-1	-1	1	4
49	56	IPA	A	-1	-1	1	-1	1	-1	1	2
50	39	IPA	A	1	-1	-1	-1	-1	1	1	3
51	8	IPA	A	-1	-1	1	1	-1	1	1	3
52	53	IPA	A	-1	1	-1	-1	1	1	1	5
53	24	MeOH	A	1	-1	1	-1	-1	-1	-1	27
54	19	MeOH	A	1	1	-1	-1	1	-1	-1	27
55	34	MeOH	A	-1	1	1	1	1	-1	-1	33
56	27	MeOH	A	-1	1	-1	1	-1	1	-1	32
57	50	MeOH	A	-1	-1	-1	1	1	1	-1	32
58	23	MeOH	A	-1	-1	-1	-1	-1	-1	1	5
59	5	MeOH	A	1	-1	1	1	-1	-1	1	2
60	44	MeOH	A	1	1	-1	1	1	-1	1	2
61	57	MeOH	A	1	1	1	-1	-1	1	1	2
62	4	MeOH	A	1	-1	1	-1	1	1	1	2
63	66	MeOH	A	-1	1	1	1	1	1	1	2
64	63	MeOH	A	0	0	0	0	0	0	0	20
65	2	MeOH	A	0	0	0	0	0	0	0	23
66	33	MeOH	A	0	0	0	0	0	0	0	19

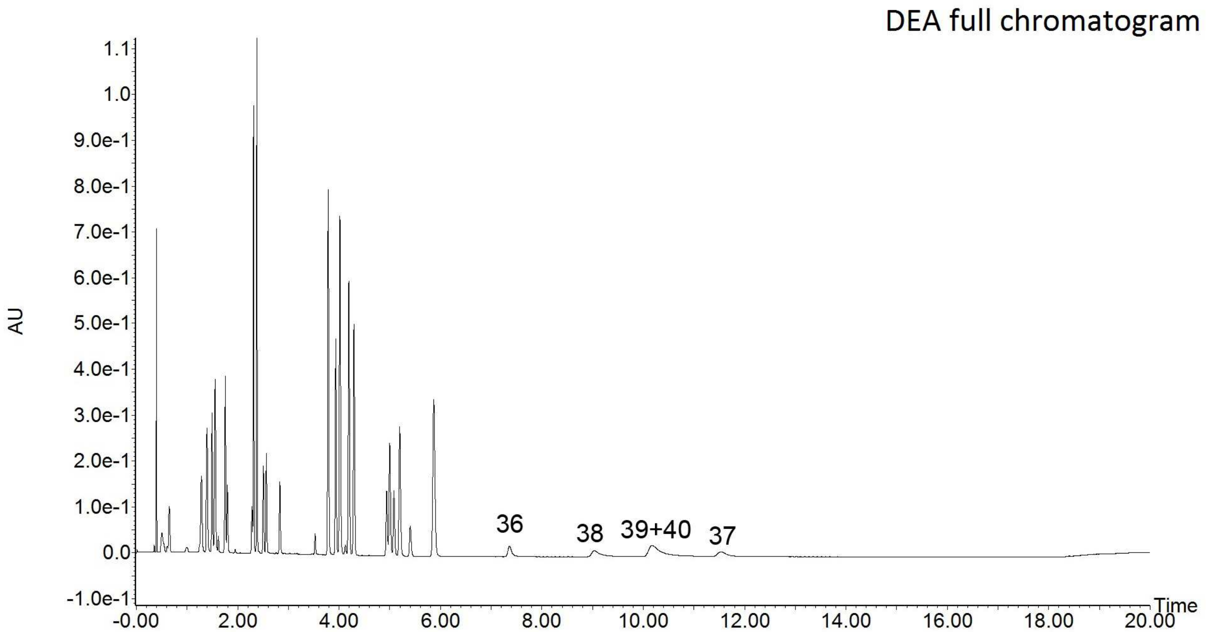

Fig. S1 Full UHPSFC-DAD chromatogram of 40 lignin-derived compounds on DEA column. For SFC condition, see "Material and method" part. For peak identities, see Table 1

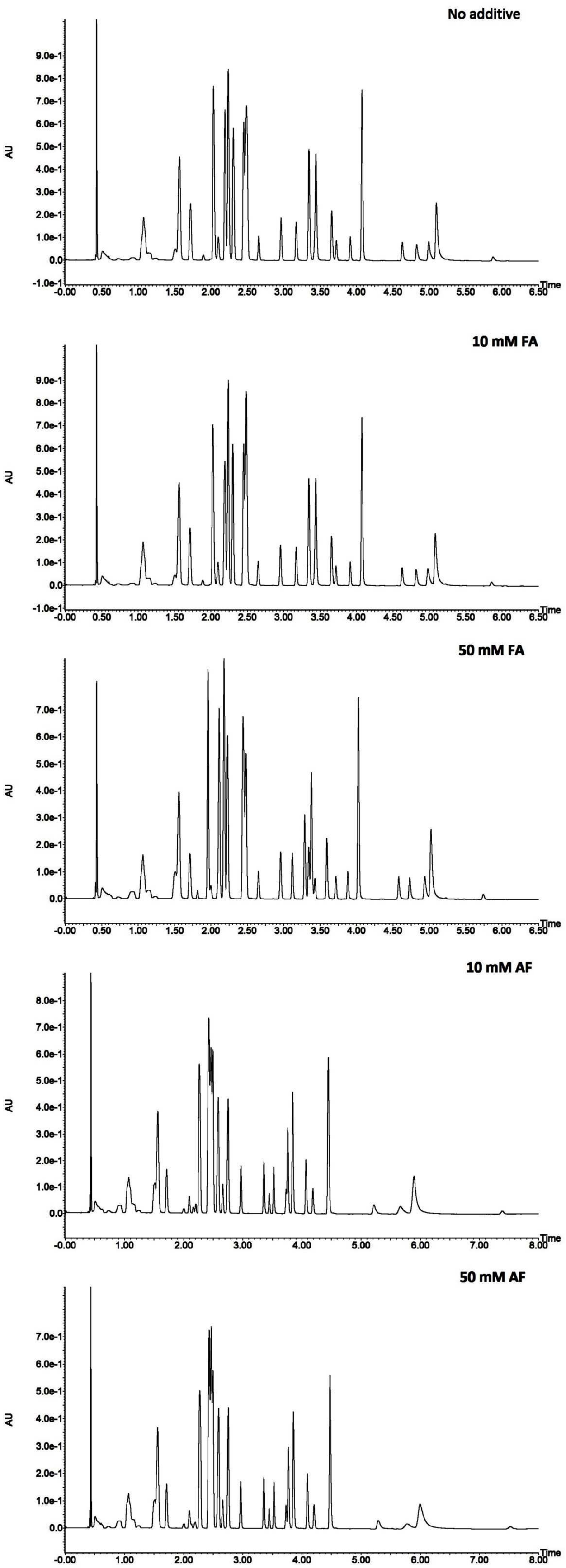

Fig. S2 The influence of different additives (formic acid and ammonium formate) to the mobile phase at different concentrations. A DIOL column was used. Experimental conditions: see Materials and methods

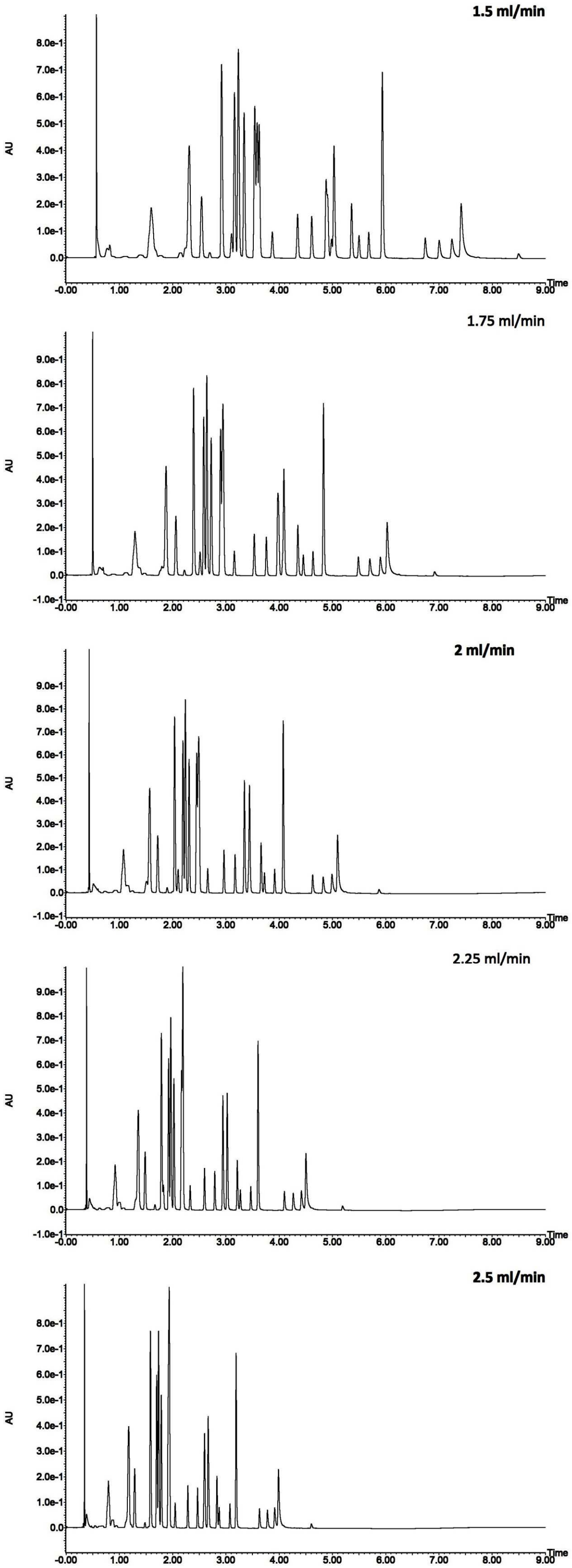

Fig. S3 The influence of different flow rates. A DIOL column was used. Experimental conditions: see Materials and methods

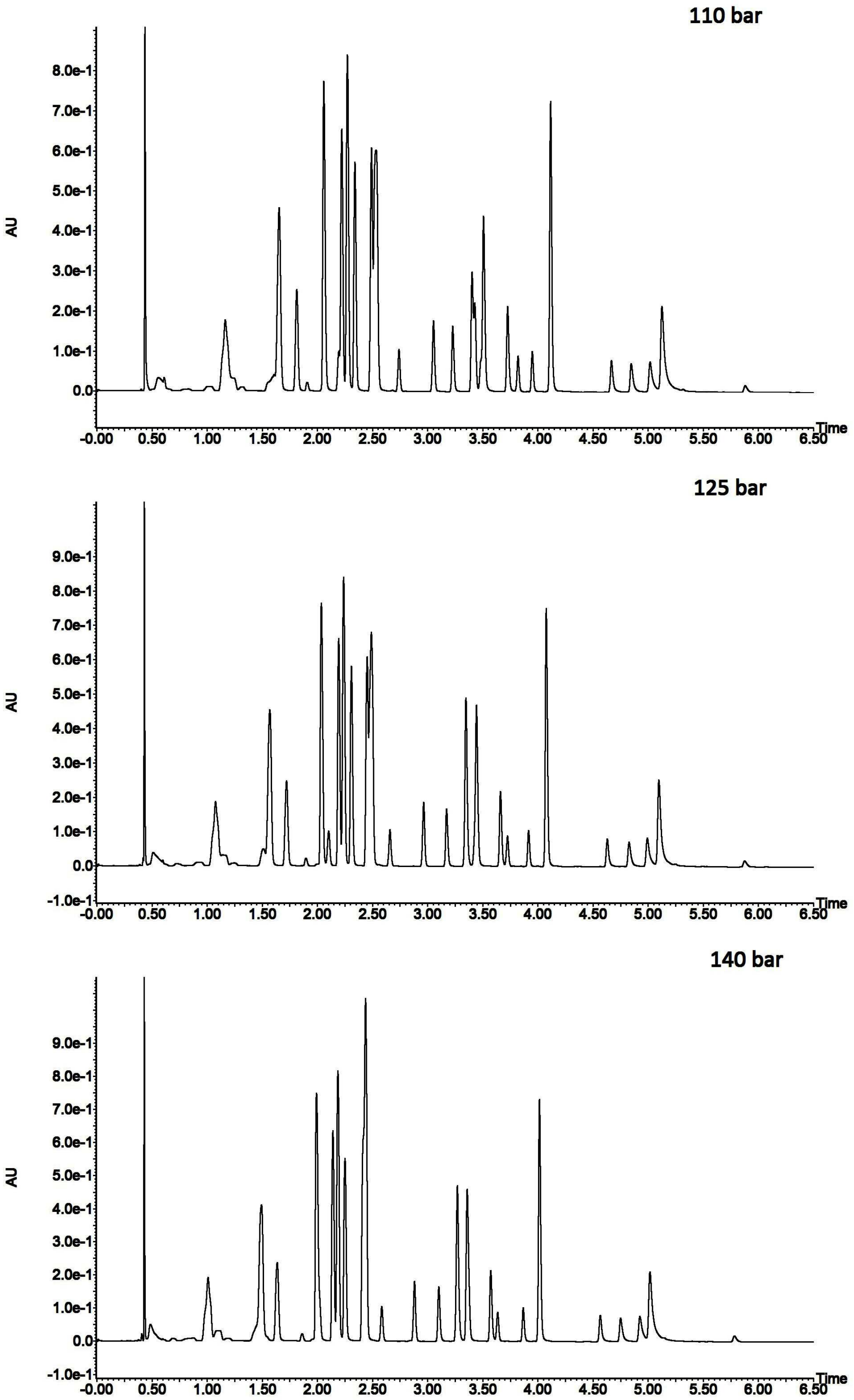

Fig. S4 The influence of different backpressure. A DIOL column was used. Experimental conditions: see Materials and methods

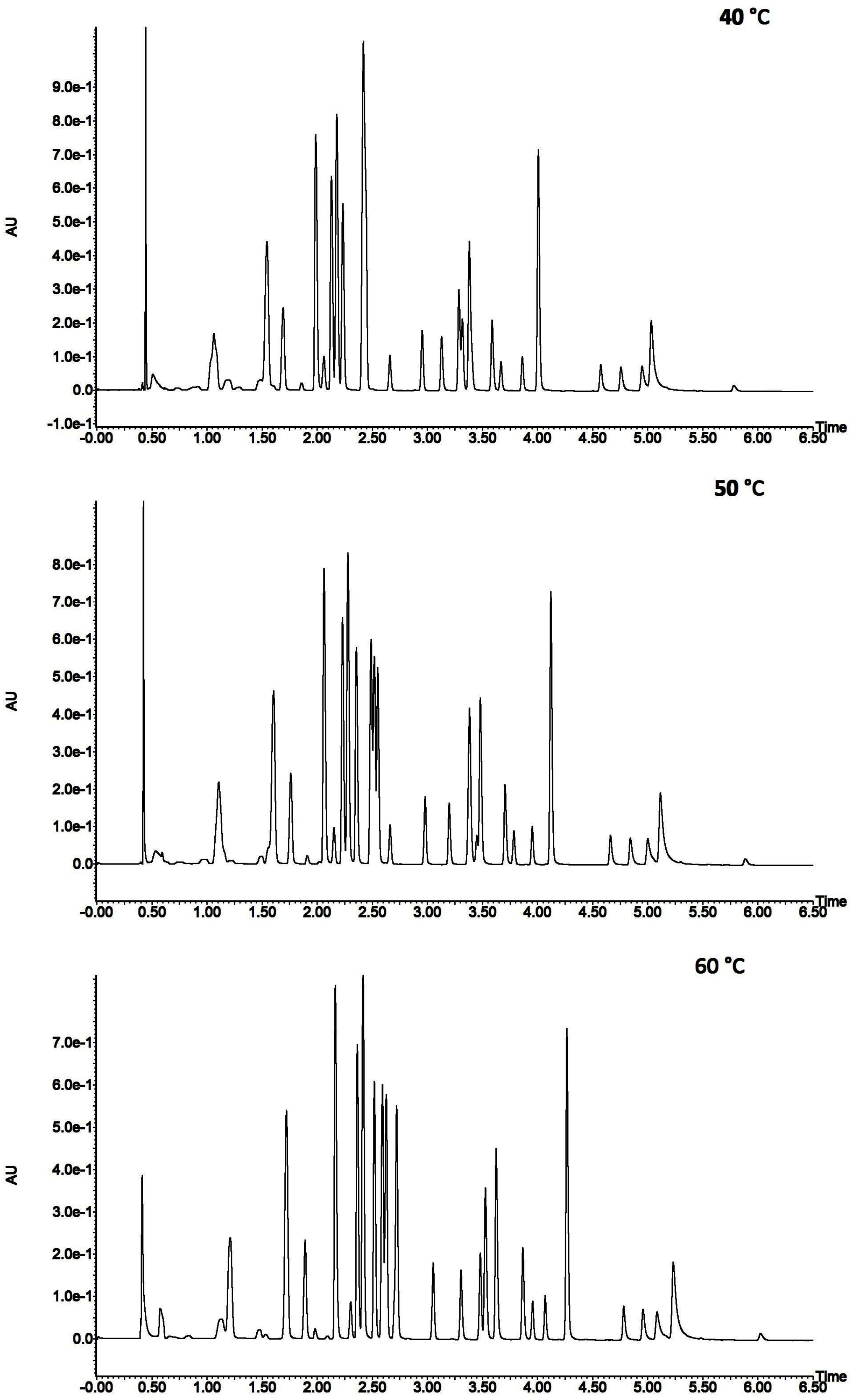

Fig. S5 The influence of different column temperatures. A DIOL column was used. Experimental conditions: see Materials and methods

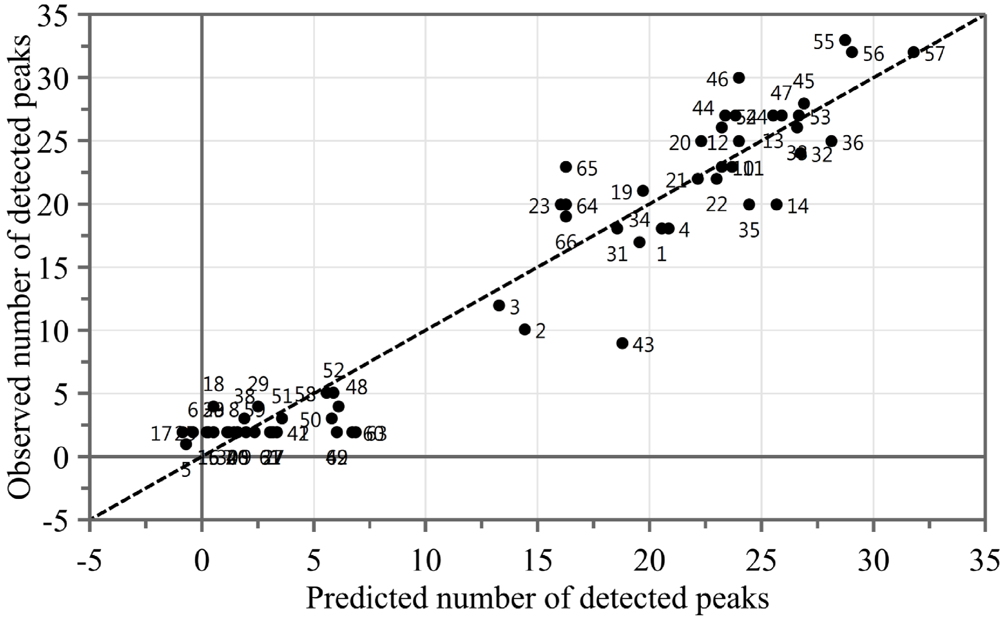

Fig. S6 Correlation between predicted and observed number of detected peaks with base peak intensity ≥1.0E5 of the D-optimal interaction model design

Information about the second design of experiment

For the more detailed investigation of the influence of the concentration of the make-up solvent additive, the desolvation gas temperature and the cone voltage a quadratic model with a face centered central composite design was created. The design had in total 17 runs including three center points. The used variable ranges are shown in Table S2 and the tested values in each experiment are shown in Table S3.

All analyses were performed in negative ionization mode, with a cone gas flow of 40 L/h and an extractor cone voltage of 4 V. Methanol was used as make-up solvent with a make-up solvent flow rate of 0.2 mL/min. A source temperature of 120 °C with a desolvation gas flow of 1200 L/h and a capillary voltage of 3.0 kV were used. The scan time was set to 0.1 sec with a scan range of m/z 50 – 1000. A BEH 2-EP column (1.7 µm, 3 mm * 100 mm) was used with a column temperature of 45 °C, a backpressure of 125 bar. The elution gradient started with 1 % B (vol. %), where it was held for 1 minute, followed by a ramp up to 25 % B (vol. %) until 9 minutes, where it was held for 1 minute, after which it returned to starting composition in 1 minute, with A being CO_2 and B methanol. The flow rate was set at 1.0 mL/min. As injection solvent methanol was used. The injection volume was set to 1.5 µL.

The optimized model showed a relative low total explained variance of 57% [$R^2(Y)$ = 0.57] and a relative low cross-validated predictability of 36% [$Q^2(Y)$ = 0.36]. The normalized influences of all variables are shown in Fig. S7. The concentration of the make-up solvent additive and the cone voltage are showing significant influence on the response. The desolvation gas temperature shows no significant influence on the response. No interactions between variables could be observed. The correlation between the predicted number of detected peaks versus the observed number of detected peaks is shown in Fig. S8. The obtained data were analyzed by linear regression. The experimental and predicted values are highly correlated ($p<0.001$) and did not differ from the ideal line with a slope of one.

Table S2 Overview of the quantitative variables for the second created design of experiment (face centered central composite design) for the more detailed investigation of the influence of the concentration of the make-up solvent additive, the desolvation gas temperature and the cone voltage on the MS ionisation efficiency of a mixture of 40 lignin-derived monomeric compounds

**Quantitative variables**	**Variables ranges**		
	**-1**	**0**	**+1**
Concentration of make-up solvent additive (mmol/L)	2.5	5	7.5
ESI source desolvation gas temperature (°C)	550	600	650
ESI source cone voltage (V)	15	20	25

Table S3 Performed experiments of the second design of experiment (quadratic model with face centered central composite design). For variable ranges see Table S2. Exp No = experiment number; CoV = cone voltage; Conc = concentration of make-up solvent additive; DeT = desolvation gas temperature; BPI = base peak ion chromatogram

Exp No	Run Order	CoV	Conc	DeT	Peaks with BPI intensity ≥1.0E5
1	15	-1	-1	-1	28
2	8	1	-1	-1	33
3	11	-1	1	-1	29
4	1	1	1	-1	32
5	10	-1	-1	1	32
6	6	1	-1	1	35
7	13	-1	1	1	30
8	7	1	1	1	34
9	5	-1	0	0	31
10	12	1	0	0	31
11	4	0	-1	0	30
12	9	0	1	0	33
13	2	0	0	-1	30
14	3	0	0	1	34
15	16	0	0	0	35
16	14	0	0	0	33
17	17	0	0	0	33

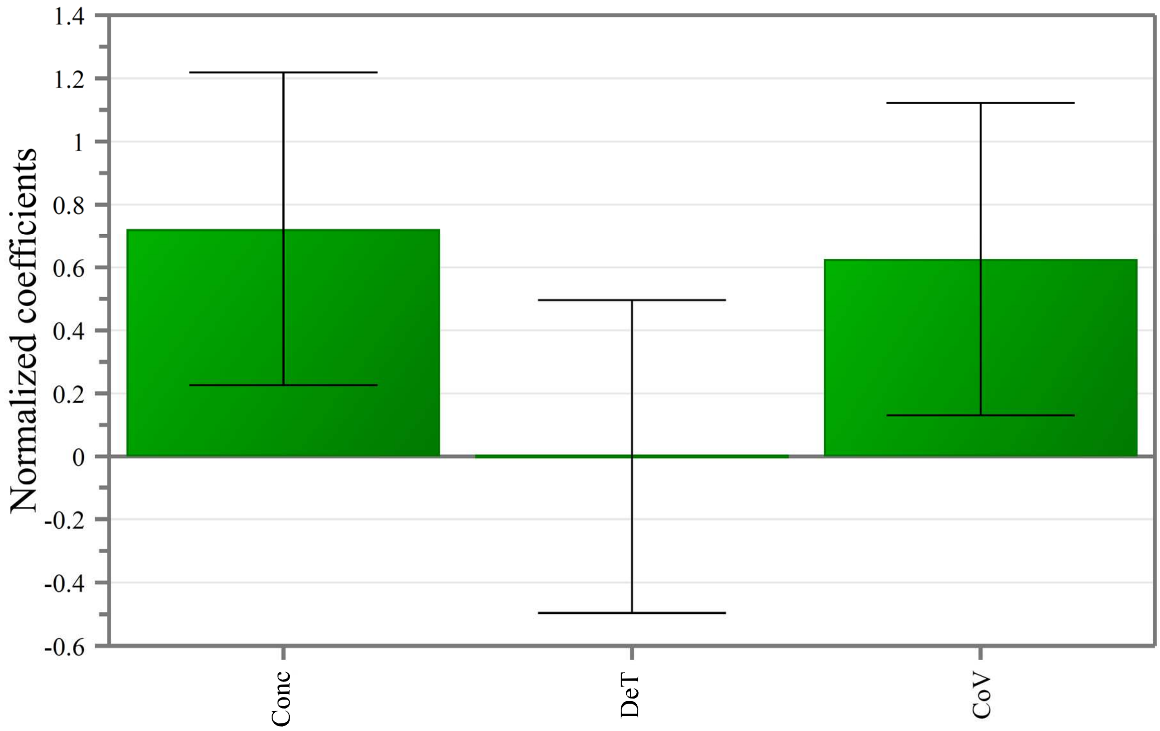

Fig. S7 Normalized influence of the investigated variables on the number of detected peaks with a base peak intensity ≥1.0E5 of the second design of experiment (quadratic model with face centered central composite design). Conc = concentration of make-up solvent additive; DeT = desolvation gas temperature; CoV = cone voltage

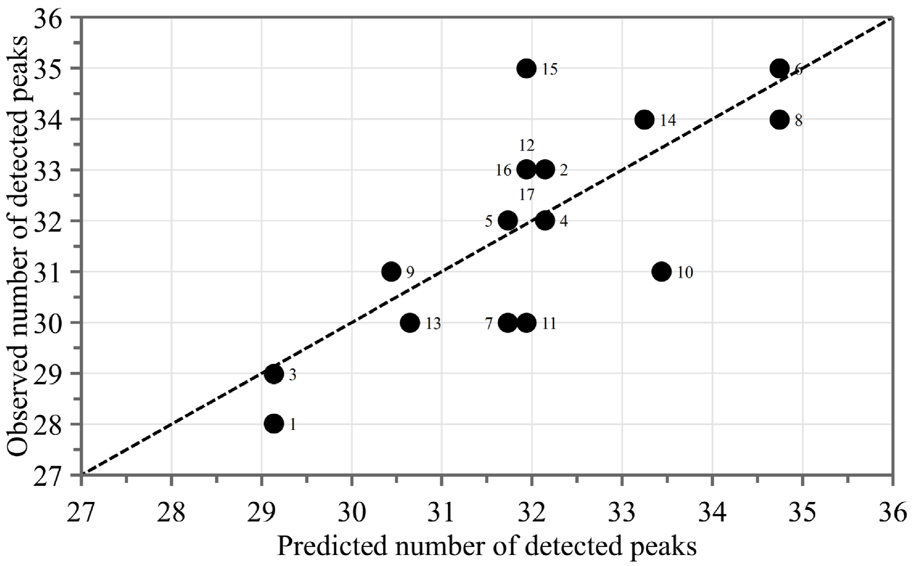

Fig. S8 Correlation between predicted and observed number of detected peaks with base peak intensity ≥1.0E5 of the second design of experiment (quadratic model with face centered central composite design)

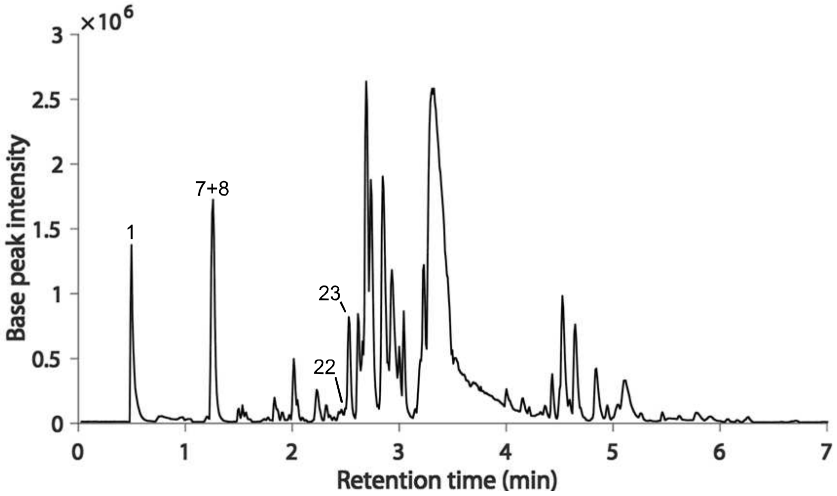

a

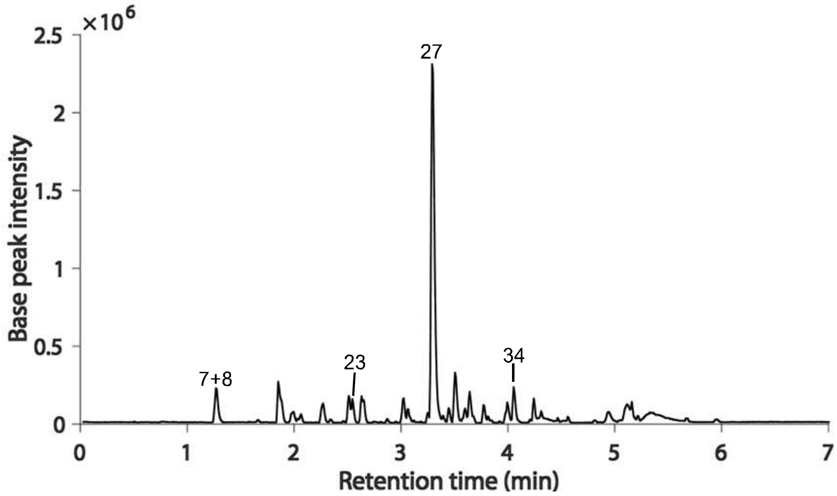

b

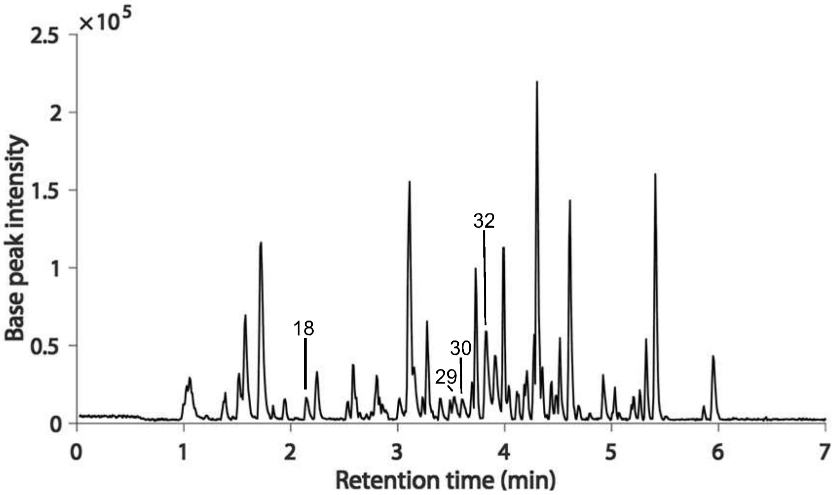

c

Fig. S9 Obtained base peak ion chromatogram for (a) lignin sample B; (b), lignin sample C; and (c) lignin sample D, using the optimized UHPSFC-QTOF/MS conditions. Experimental conditions: see Materials and methods. Identified compounds in sample B, C and D with the use of the multi-standard are labeled according to the compound number in Table 1
